# Sex ratio at mating does not modulate age fitness effects in *Drosophila melanogaster*

**DOI:** 10.1002/ece3.5227

**Published:** 2019-05-21

**Authors:** Zahida Sultanova, Pau Carazo

**Affiliations:** ^1^ Behaviour & Evolution Group, Ethology Lab, Cavanilles Institute of Biodiversity and Evolutionary Biology University of Valencia Valencia Spain

**Keywords:** aging, fitness, reproductive success, sex ratio, social context

## Abstract

Understanding the effects of male and female age on reproductive success is vital to explain the evolution of life history traits and sex‐specific aging. A general prediction is that pre‐/postmeiotic aging processes will lead to a decline in the pre‐ and postcopulatory abilities of both males and females. However, in as much the sexes have different strategies to optimize their fitness, the decline of reproductive success late in life can be modulated by social context, such as sex ratio, in a sex‐specific manner. In this study, we used *Drosophila melanogaster* to investigate whether sex ratio at mating modulates age effects on male and female reproductive success. As expected, male and female age caused a decrease in reproductive success across male‐biased and female‐biased social contexts but, contrary to previous findings, social context did not modulate age‐related fitness decline in either of the two sexes. We discuss these results in the light of how sex ratio might modulate pre‐/postcopulatory abilities and the opportunity for inter‐ and intrasexual competition in *D. melanogaster*, and generally suggest that social context effects on these processes are likely to be species specific.

## 1 INTRODUCTION

Exploring the effects of male and female age on reproductive success is vital to understand life history evolution and sex‐specific aging (Bonduriansky, Maklakov, Zajitschek, & Brooks, 2008; Flatt & Heyland, 2011; Maklakov & Lummaa, 2013; Zajitschek, Bonduriansky, Zajitschek, & Brooks, 2009). Both male and female reproductive success is expected to decrease with age due to a functional decline in pre‐ and postcopulatory reproductive processes and/or in other phenotypic traits with indirect effects on reproductive success, such as exploratory activity, locomotion, perception, or immunity (Monaghan, Charmantier, Nussey, & Ricklefs, 2008; Nussey, Froy, Lemaitre, Gaillard, & Austad, 2013; Pizzari, Dean, Pacey, Moore, & Bonsall, 2008; Reznick, Bryant, Roff, Ghalambor, & Ghalambor, 2004). Many studies have explored how female age can reduce fertility and fecundity (David, Cohet, & Fouillet, 1975; Deng, 2012; Holmes, Thomson, Wu, & Ottinger, 2003), or how male age can decrease copulation success, fertilizing ability, and sperm competition (Service & Fales, 1994; Economos, Miquel, Binnard, & Kessler, 1979; Kühnert & Nieschlag, 2004). More recent studies have further investigated how female and male age can decrease offspring viability by focusing on underlying pre‐ and postmeiotic aging processes (Firman, Young, Rowe, Duong, & Gasparini, 2015; Pizzari et al., 2008; Tan, Pizzari, & Wigby, 2013). In short, the effects of age on reproductive success have been relatively well explored, yet most available studies have overlooked how socio‐sexual factors such as density or sex ratio might modulate reproductive senescence across the sexes (Brengdahl, Kimber, Maguire‐Baxter, Malacrinò, & Friberg, 2018; Carazo, Molina‐Vila, & Font, 2011; Fricke, Green, Mills, & Chapman, 2013; Ruhmann, Koppik, Wolfner, & Fricke, 2018; Tan et al., 2013; Zhao, Xuan, Li, & Xi, 2008).

The social context (e.g., sex ratio and density) can modulate age effects on reproductive success in a sex‐specific way by influencing different factors such as mate encounter rate, mate choice, or intrasexual competition (Kokko & Rankin, 2006; Kvarnemo & Ahnesjo, 1996). For example, in the feral fowl, a species with strong male–male competition over female harems, Dean et al. (2010) found that old males had the potential to sire a relatively higher proportion of offspring in groups with a female‐biased (FB) sex ratio, compared to a male‐biased (MB) sex ratio. This was due to old males having a higher chance of being socially dominant in FB groups, where male–male competition was low (Dean et al., 2010). Unfortunately, this interesting result has not been followed up by similar studies in other organisms with different mating systems, nor with respect to female age.

In this study, we used *Drosophila melanogaster* to explore how male and female age affects the reproductive success of males and females in experimental mating patches with FB or MB sex ratios. In *D. melanogaster*, males have strong intrasexual competition over mating and allocate considerable time and effort to court available females, while females are able to re‐mate with multiple males (Dow & Schilcher, 1975; Markow, 2002; Pitnick, 1991). Although intrasexual competition is mostly observed in males, aggression between females is also present, mainly when food sources are scarce (Bath et al., 2017; Bath, Morimoto, & Wigby, 2018; Ueda & Kidokoro, 2002). Furthermore, both males and females of this species exhibit mate choice but the degree and direction of these choices can differ depending on the population of origin and the social environment (Byrne & Rice, 2006; Edward & Chapman, 2012, 2013; Gowaty, Steinichen, & Anderson, 2003; Monier, Nöbel, Isabel, & Danchin, 2018). Based on its mating system, we predicted that sex ratio would modulate age effects on the fitness of males and females differently. In males, intrasexual competition is believed to be stronger than intersexual selection (Gowaty et al., 2003). Hence, we predicted that male age would decrease reproductive success relatively more in a MB social context, because we expected old males to have a higher disadvantage under intense male–male competition. In contrast, in this species female intrasexual competition appears to be less important than intersexual selection (Gowaty et al., 2003), so we did not predict a similar outcome. Instead, *D. melanogaster* males exhibit a marked preference for young females (Cook & Cook, 1975; Lüpold, Manier, Ala‐Honkola, Belote, & Pitnick, 2011), so we predicted female age to decrease reproductive success more in a FB social context, where there is a potentially higher opportunity for males to choose young females over the old ones.

## 2 METHODS

### 2.1 Experimental populations

We used *D. melanogaster* flies from a laboratory‐adapted, wild‐type Dahomey stock population derived from an original population founded in 1970 (Partridge & Farquhar, 1983). Our population is maintained with overlapping generations at 25°C, ~50%–60% humidity and a 12 hr:12 hr Light:Dark cycle, and fed with a diet that contains yeast (10 g/L), sugar (50 g/L), soy flour (10 g/L), corn flour (60 g/L), nipagin (3 g/L) and 0.05% propionic acid. To obtain experimental flies, we collected eggs from our population cages using grape‐agar filled petri dishes with a smear of live yeast paste, at standardized density (Clancy & Kennington, 2001). We collected virgin adults emerging from these eggs within 7 hr of eclosion and generated old focal males and females by isolating them with excess food for 28 days prior to assays, during which time we flipped them into a new vial once a week. In contrast, young focal males and females were only kept in isolation for 3 days after their emergence and prior to assays. Young *sparkling (spa)* competitors/partners were kept in same‐sex groups of 10 for 3 days after their emergence and until the beginning of assays. Using *spa* flies as competitors, which are homozygous for the recessive *spa* allele and exhibit a rough‐eye phenotype, allowed us to differentiate between the offspring of wt and *spa* parents in competitive fitness assays (e.g., Fricke, Wigby, Hobbs, & Chapman, 2009).

### 2.2 Competitive fitness assays

**Figure 1:**
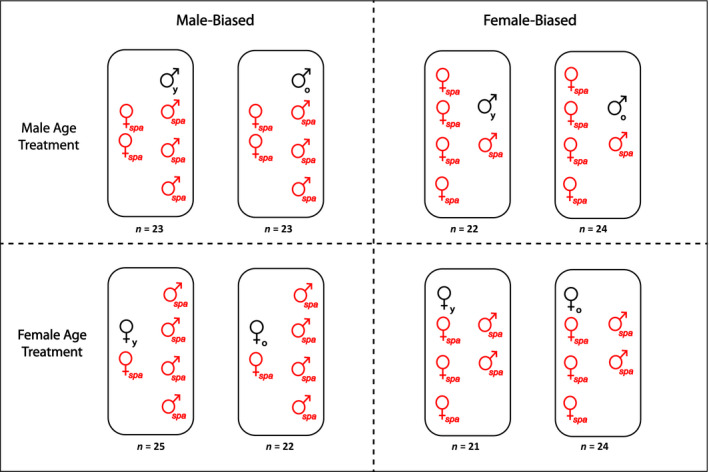
Scheme of the different treatments implemented to measure the reproductive success of young (y) and old (o) focal flies across different sex ratios

In order to explore the effect of sex ratio and age on reproductive success, we studied the fitness of focal wt male and female flies when competing against *spa* rivals, in a factorial combination of sex ratio (i.e., MB—four males and two females—vs. FB—four females and two males–) and age (i.e., a young vs. old focal wt male/female competing against young *spa* rivals for young *spa* mating partners). Thus, within each vial, all flies except the focal experimental fly were *spa* (Figure 1). For all treatments, we allowed flies to interact and lay eggs for 2 days, after which time we discarded the males and allowed females to oviposit for three more days in a fresh vial. In order to control for larval density across treatments during this second period of oviposition, we separated the four females in FB sex ratio treatments in two vials containing two females each. After transferring/discarding females, we incubated vials from both the first and second period of oviposition for 16 days, froze the vials, and then proceeded to count the number of *spa* and wt offspring in each vial. In order to control for the potential effects of density on the development of larvae from eggs laid during the first oviposition period, we counted the number of pupae in these vials. The density of larvae per vial (number of pupae ± *SEM* = 44.7 ± 1.0) was, in all cases, comfortably below the threshold for which density effects have been described in *D. melanogaster* (Miller & Thomas, 1958)).

### 2.3 Statistical analysis

To determine the effect of aging and sex ratio on reproductive success in a way that is comparable across the two different sex ratio treatments (i.e., fixed density but which include a different amount of males and females), we first standardized data. We calculated the standardized reproductive success of each focal female by subtracting the average number of offspring that belong to competitors (*spa*) from the observed number of offspring that belong to the focal fly (wt) for each replicate:

$$\#\;\text{of}\;\text{offspring}\;\text{from}\;\text{focal}-\frac{\#\;\text{of}\;\text{offspring}\;\text{from}\;\text{competitors}}{\#\;\text{of}\;\text{competitors}}$$

We calculated the standardized reproductive success of each focal male using the same equation, but divided by the number of females that were present in the corresponding mating vial (two females in MB social context and four females in FB social context).

After standardization, we checked for heteroscedasticity and normality via graphical exploration, and subsequently validated all models. To explore the effect of age and sex ratio on reproductive success of each sex separately, we fitted a linear model including age, sex ratio, and their interactions as fixed factors. We then repeated this analysis using a restricted maximum likelihood LMMs (Linear Mixed Models) approach and introducing pupae density as a random intercept effect. In order to obtain minimum adequate models, we performed backward stepwise model selection based on Likelihood Ratio Tests (LRTs). All analyses were performed in R v. 3.3.2 (R Core Team, 2016).

## 3 RESULTS

**Figure 2:**
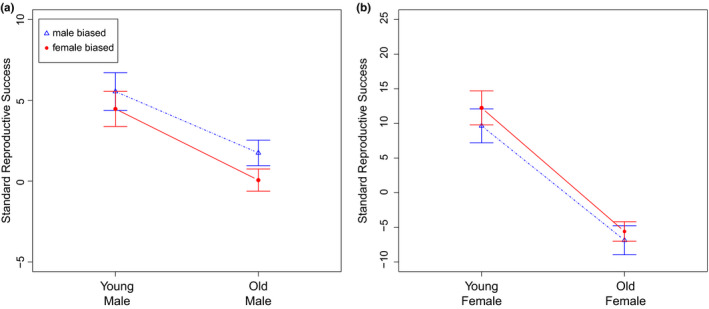
Standard reproductive success of (a) young/old focal males in male‐biased and female‐biased social contexts and (b) young/old focal females in male‐biased and female‐biased social contexts

When we did not control for pupae density, we did not find a significant age × sex ratio interaction (*F*_1,88_ = 0.1027, *p* = 0.7494) or sex ratio effect (*F*_1,89_ = 2.1731, *p* = 0.144) in male reproductive success. However, we found a significant main effect of age (*F*_1,89_ = 19.2600, *p* < 0.001, Figure 2a). Similarly, in the case of female reproductive success, we did not find a significant age × sex ratio interaction (*F*_1,88_ = 0.0967, *p* = 0.7566) or sex ratio effect (*F*_1,89_ = 0.8208, *p* = 0.3674) but we did find a significant age effect (*F*_1,89_ = 64.1757, *p* < 0.001, Figure 2b).

Controlling for pupae density did not qualitatively change our results. For males, there was no significant age × sex ratio interaction (χ2 = 0.1073, *df* = 1, *p* = 0.7433) or sex ratio effect (*F*_1,89_ = 2.1731, *p* = 0.144); whereas, we did find a significant age effect (*F*_1,89_ = 18.9707, *p* < 0.001) on male reproductive success. Similarly, for females, we did not find a significant age × sex ratio interaction (χ2 = 0.101, *df* = 1, *p* = 0.7506) or sex ratio effect (*F*_1,89_ = 0.8140, *p* = 0.3674). However, we found a significant main effect for age (*F*_1,89_ = 64.8510, *p* < 0.001).

## 4 DISCUSSION

In this study, we investigated the potential role that sex ratio at mating might play in modulating the fitness effects of age in *D. melanogaster* males and females. We found that both male and female age caused a decline in reproductive success but, contrary to our expectations, this effect was not modulated by sex ratio at mating (i.e., was similar in both MB and FB social context).

In the case of males, we were expecting male age to decrease the reproductive success more in a MB social context, where intrasexual competition is high. Like in many other organisms, in *D. melanogaster* male–male competition is expected to increase drastically in MB social contexts (Wang & Anderson, 2010). In principle, this should lead to old males having relatively higher reproductive fitness in FB contexts, where male–male competition is low. Accordingly, Dean et al. (2010) showed that, in the feral fowl (*Gallus gallus*), the effects of age on the reproductive success of males were mitigated in FB (vs. MB) contexts. In this species, socially dominant males have privileged access to mating opportunities but females mate multiply, so sperm competition is intense (David Ligon & Zwartjes, 1995; Dean et al., 2010; Pizzari & Birkhead, 2000; Pizzari, Froman, & Birkhead, 2002). Dean et al. (2010) elegantly showed that despite old males having a lower sperm competition ability than young males, they had a relative advantage in FB (vs. MB) social groups, due to a higher possibility of being socially dominant when male–male intrasexual competition is low. Our failure to find similar effects in *D. melanogaster* may have to do with inherent differences in the mating system of these two species.

In fruit flies, male–male competition over access to females is generally high and, in the wild, males seem to exhibit a typical resource‐defense polygyny by defending pieces of decaying fruit where females feed (Markow, 1988). Recent evidence suggests that male–male aggression in this context also serves a mate‐guarding function (Baxter, Barnett, & Dukas, 2015), but flies do not live in stable social groups and hence males cannot monopolize access to females throughout their lifespan. Furthermore, laboratory populations like the one used in this study have been kept at high densities for thousands of generations, in conditions where mate monopolization is highly unlikely. As a result, the level of intrasexual competition in this species might not modulate age‐related fitness effects as it does in feral fowls (or might do so to a lesser extent). On the other hand, intersexual competition also seems to be quite important in *D. melanogaster*, and there is good evidence that both female and male mate choice are modulated by social context (Edward & Chapman, 2012, 2013; Monier et al., 2018). In particular, females prefer mating with young (or large) males that court more vigorously (Jagadeeshan, Shah, Chakrabarti, & Singh, 2015; Rezaei, Krishna, & Santhosh, 2015), and they appear to be less choosy when sex ratios are FB (Monier et al., 2018). Hence, old males might be expected to benefit in FB contexts due to females being less choosy in favor of young males. This makes it more striking that we did not find sex ratio to modulate age effects on male reproductive success.

It is possible that our results for males are partly explained by male mate choice effects. Under FB sex ratios, where males are expected to be choosier, young males may benefit by choosing high‐quality females while old males are left to mate with low‐quality females. In *D. melanogaster,* aging seems to diminish the ability of males to choose high‐quality females (Hu, Han, Wang, & Xue, 2014), so that old males may fail to be choosy despite ample opportunity for male mate choice in FB contexts, to the benefit of young “choosy” males. An intriguing possibility is that mate choice copying (Nöbel, Danchin, & Isabel, 2018) may have contributed to exacerbate male age effects in the FB sex ratio. In *D. melanogaster*, females prefer mating with young males that court more vigorously (Jagadeeshan et al., 2015; Rezaei et al., 2015) and, in our experiment, old and young focal males were always phenotypically distinguishable to their young rival sparkling flies (i.e. different eye‐color). Given recent findings showing that females tend to copy the mate choice of other females based on male color cues in fruit flies (Danchin et al., 2018), it is possible that the inherent advantage of young males over old males due to female choice may have been exacerbated in the FB context, where mate choice copying is more likely. In short, young males may hold a similar fitness advantage against old males irrespective of the sex ratio, but via different sexual selection mechanisms: via intrasexual competition and female mate choice, when the sex ratio is MB, and via male mate choice and female mate copying when the sex ratio is FB.

In the case of females, female reproductive success also decreased with age similarly in both FB and MB social contexts (Figure 2b). We might have expected that in a FB social context with a higher opportunity for males to be choosy, males (which are all young in this case) would prefer to mate with young (vs. old) focal females, which would thus have higher reproductive success. Several previous studies have reported the existence of both pre and postcopulatory male mate choice with respect to female age. For example, male courtship intensity decreases with female age (Cook & Cook, 1975) and males allocate less sperm to old females compared to their young counterparts (Lüpold et al., 2011). However, being attractive to males is not always beneficial for females. Mating and male harassment are known to decrease survival and reproductive success in female *D. melanogaster* (Chapman & Partridge, 1996; Partridge & Fowler, 1990; Partridge, Green, & Fowler, 1987; Wigby & Chapman, 2005). The fact that male preference for young females may have been more marked in the FB social context could have led males to be more harmful to these females, which in turn may have counterbalanced any benefits from male mate choice. As a matter of fact, Long, Pischedda, Stewart, and Rice (2009) showed that male harm is preferentially directed toward intrinsically higher‐fitness females and that, as a result, any fitness advantage that could be experienced by high condition females (young females in our design) might be compensated by the costs of being attractive in a FB social context (at least in simple environments such as the one used in this experiment; see Yun, Chen, Singh, Agrawal, & Rundle, 2017; MacPherson, Yun, Barrera, Agrawal, & Rundle, 2018). Similarly, relatively high mating costs in a MB social context might also contribute to explain why we did not observe a sex ratio × female age interaction. Although the opportunity for male mate choice is lower in this context and males might thus harm both young and old females, mating costs may be expected to be more pronounced in old females, which would tend to exacerbate age effects in a MB social context. Unfortunately, we currently have very little information about how social context changes intra‐ versus intersexual competition in males and females, in *D. melanogaster* or other species, which means the above possibilities remain to be explored.

Studies of reproductive senescence so far have focused on understanding the effect of male and female age on reproductive success (Flatt & Heyland, 2011; Williams, 1957), for example by studying male/female age effects on pre–post copulatory mating abilities, mate choice, and offspring viability (Carazo et al., 2011; Cook & Cook, 1975; Dunson, Baird, & Colombo, 2004; Lüpold et al., 2011; Maklakov et al., 2009; Tan et al., 2013; Velando, Noguera, Drummond, & Torres, 2011). Many studies have also investigated the interaction between social context and several fitness traits such as mating duration, reproductive success, survival, and lifespan (Adler & Bonduriansky, 2011; Bretman, Fricke, Hetherington, Stone, & Chapman, 2010; Bretman, Westmancoat, Gage, & Chapman, 2013; Costa, Mateus, Moura, & Machado, 2010; Iliadi, Iliadi, & Boulianne, 2009; Leech, Sait, & Bretman, 2017; Zajitschek, Zajitschek, Friberg, & Maklakov, 2013). In sharp contrast, how age effects on reproductive success may be modulated by the social context has so far been largely overlooked even though social context (such as sex ratio at mating) might play a crucial role in modulating sex‐specific age effects on reproductive success. We suggest future studies should aim to fill this gap in knowledge.

## CONFLICT OF INTEREST

None declared.

## AUTHORS CONTRIBUTION

ZS and PC conceived and designed the study; ZS carried out the experiment, analyzed the data and wrote the manuscript with the revisions of PC; ZS and PC revised and approved the final form of the manuscript.

## Data Availability

Data is archived in the DRYAD data repository, the doi for our data is https://doi.org/10.5061/dryad.s67v7b1.
